# Volatile Organic Compounds of *Scheffersomyces spartinae* W9 Have Antifungal Effect against *Botrytis cinerea* on Strawberry Fruit

**DOI:** 10.3390/foods12193619

**Published:** 2023-09-28

**Authors:** Xiurong Zou, Yingying Wei, Jianhua Zhu, Jincai Sun, Xingfeng Shao

**Affiliations:** 1 College of Food Science and Technology, Guangdong Provincial Key Laboratory of Utilization and Conservation of Food and Medicinal Resources in Northern Region, Shaoguan University, Shaoguan 512005, China; 2 State Key Laboratory for Managing Biotic and Chemical Threats to the Quality and Safety of Agro-Products, Zhejiang-Malaysia Joint Research Laboratory for Agricultural Product Processing and Nutrition, College of Food and Pharmaceutical Sciences, Ningbo University, Ningbo 315800, China; 3 Faculty of Food Science, Zhejiang Pharmaceutical University, Ningbo 315500, China

**Keywords:** antagonistic yeast, biocontrol, gray mold, volatile organic compounds (VOCs), 2-phenylethanol

## Abstract

This study aims to evaluate the antifungal effects of volatile organic compounds (VOCs) produced by a marine biocontrol yeast, *Scheffersomyces spartinae* W9. The results showed that the VOCs from the yeast inhibited the growth of *Botrytis cinerea* mycelium and spore germination by 77.8% and 58.3%, respectively. Additionally, it reduced the disease incidence and lesion diameter of gray mold on the strawberry fruit surface by 20.7% and 67.4%, respectively. Electronic micrographs showed that VOCs caused damage to the morphology and ultrastructure of the hyphae. Based on headspace solid-phase microextraction gas chromatography–mass spectrometry (HS-SPME/GC-MS), *S. spartinae* W9 emitted 18 main VOCs, and the pure substance of VOCs, such as 3-methyl-1-butanol, 2-methyl-1-butanol, 2-phenylethanol, and isoamyl acetate, showed antifungal effects against *B. cinerea* mycelium growth. Among them, 2-phenylethanol exhibited the strongest antifungal activity. It has been concluded that VOCs are the key antifungal mechanism of *S. spartinae* W9, and a promising strategy for controlling gray mold on strawberry fruit.

## 1. Introduction

So far, a lot of antagonistic yeasts have been discovered to suppress fruit and vegetable diseases caused by phytopathogenic fungi, such as gray mold, green mold, brown rot, and root rot [1]. The biocontrol mechanisms exhibited by antagonistic yeasts are diverse and depend on species to species. They primarily include competition for nutrition and space, parasitism, biofilm formation, production of diffusible or volatile antimicrobial substances, and induction of plant host resistance [1,2]. 

During recent years, volatile organic compounds (VOCs) have been proven to be one of the most crucial biocontrol mechanisms of antagonistic yeasts [2]. VOCs can not only inhibit the mycelial growth and spore germination of pathogenic fungi, but also limit the production of mycotoxins. For instance, Farbo et al. [3] found that VOCs produced by yeast remarkably suppressed the growth, sporulation, and ochratoxin A production in *Aspergillus* spp. Furthermore, yeast VOCs have demonstrated efficacy in controlling gray mold in strawberry fruit [4], green mold and blue mold in mandarin fruit [5], green mold in lemons [6], and brown rot in cherry, peach, and apricot fruit [7]. Notably, yeast VOCs have also been found to enhance the disease resistance of plants. Di Francesco et al. [7] observed an increase in the concentration of membrane proteins, as well as the biosynthesis of cuticle and wax, in stone fruits after treatment with VOCs derived from *Aureobasidium pullulans*. They speculated that VOCs enhanced the disease resistance by changing the fruit’s structural composition. 

VOCs refer to a category of organic compounds with a molecular weight of less than 300 Da, high vapor pressure, low boiling point, and low water solubility [8]. They mainly include alcohols, ketones, aldehydes, phenols, acids, benzene rings, heterocycles, mercaptans, and thioesters [9]. The volatility of VOCs allows them to diffuse in soil particles and the air, enabling them to function without direct physical contact with pathogenic microorganisms. The compound of VOCs varies with the yeast species, the presence of other microorganisms, culture time, nutritional conditions, ambient temperature, etc. [10]. Nasanit et al. [11] found that the emitted VOCs were distinct among five different species of antagonistic yeast, and even among different strains of the same species, the produced VOCs varied. Due to their individual and complicated content, it is vital to explore the composition, functions, and mechanisms of biocontrol yeast VOCs for their further practical application. 

In a previous study, a strain of antagonistic yeast, *Scheffersomyces spartinae* W9, was isolated from a tropical marine sea. It has exhibited excellent biocontrol effects against *Botrytis cinerea*, a common fungal pathogen of stored fruit and vegetables [12], both in vitro and in strawberry fruit, and the VOCs produced by *S. spartinae* W9 have been found to inhibit the growth of *B. cinerea* mycelium [13]. However, there have been limited reports on the biocontrol abilities of *S. spartinae* W9 and its VOCs. Therefore, this study aims to further explore the antifungal effects of VOCs secreted by *S. spartinae* W9 on stored strawberry fruit. Specifically, this study includes four parts: (i) evaluating the biocontrol effects of VOCs on the mycelium growth and spore germination of *B. cinerea*, as well as gray mold in strawberry fruit; (ii) analyzing the effects of VOCs on the morphology and ultrastructure of *B. cinerea* mycelium; (iii) determining the composition of VOCs; and (iv) investigating the antifungal effects of the main substances present in the VOCs.

## 2. Materials and Methods

### 2.1. Fruit

Strawberry (*Fragaria ananassa* Duch. cv. Hongyan) fruit was picked early in the morning from a local orchard in Ningbo, China. All fruits were of similar maturity (with uniform color and size). The fruits were free from any mechanical, pathological, or pest damage.

### 2.2. Pathogen and the Yeast

*B. cinerea* (strain No. ACCC 36028) was purchased from the Agricultural Culture Collection of China in Beijing and stored in glycerol at −80 ℃. To rejuvenate *B. cinerea*, it was inoculated on potato dextrose agar medium (PDA, 20% fresh potato, 2% glucose, 2% agar). Then, the plates were incubated at 25 °C for 5 d, and this process was repeated twice before use. 

To prepare the spore suspension of *B. cinerea*, a 10-day-old *B. cinerea* culture was rinsed with sterile distilled water. The resulting liquid was filtered through two layers of sterile 300-mesh silk cloth to remove mycelia. The concentration of *B. cinerea* spore suspension was then adjusted at 1 × 10^5^ spores mL^−1^ using a hemocytometer.

*S. spartinae* W9 was isolated from marine sediment and identified through molecular biology analysis in our previous study [13]. To prepare the yeast suspension, a ring of yeast colony from a glycerol tube stored at −80 °C was selected. The colony was then inoculated into nutrient yeast dextrose broth (NYDB, 0.8% beef paste, 0.5% yeast extract, 1% glucose). The culture was incubated on a shaker at 28 °C and a shaking speed of 180 rpm for 48 h. After one subculture, the culture medium was centrifuged to obtain yeast pellets. The pellets were rinsed twice with sterile distilled water. Finally, the yeast pellets were suspended again in sterile distilled water, with the concentration adjusted to 1 × 10^8^ cells mL^−1^.

### 2.3. Effects of VOCs Produced by S. spartinae W9 on Mycelium Growth and Spore Germination of B. cinerea

In a dual culture assay, the effects of VOCs produced by *S. spartinae* W9 on pathogen mycelium growth were assessed, following the method described by Zou et al. [14]. Briefly, a 100 μL yeast suspension of *S. spartinae* W9 (1 × 10^8^ cells mL^−1^) was inoculated and spread evenly on plates of nutrient yeast dextrose agar (NYDA: 0.8% beef paste, 0.5% yeast extract, 1% glucose, 2% agar). An equal volume of sterile distilled water was used as a control. The yeast plates were then incubated at 28 °C for 48 h. 

Mycelium growth evaluation: a 9 mm mycelium block, obtained from a 5-day-old culture of *B. cinerea*, was laid on a PDA plate. The NYDA plate previously inoculated with *S. spartinae* W9 was placed on the top of the PDA plate. The two based plates were sealed with parafilm and incubated at 25 °C for 5 d. At the end of the incubation period, the diameter of *B. cinerea* colony was measured. Each treatment had three biological replicates, and the experiment was repeated twice.

Spore germination assessment: a spore suspension (1 × 10^5^ spores mL^−1^, 1 mL) was pipetted into a 3.5 cm sterile petri dish. The dish’s cover was removed, and the dish was placed in the center of an NYDA plate previously incubated with *S. spartinae* W9 for 48 h. The dish was then sealed and incubated at 25 °C for 14~19 h. An NYDA plate inoculated with sterile distilled water served as a control. The spore germination per 100 spores was observed with a 40× optical microscope. Conidia germination was recorded when the length of the germ tube reached or exceeded half the diameter of the conidia. The spore germination rate (%) = number of germinated conidia/total number of conidia × 100. Each treatment was replicated three times, and the experiment was conducted two times.

### 2.4. Effect of VOCs on Strawberry Gray Mold

A 3 × 3 mm wound was pricked at the equator of strawberry fruits using a sterile iron nail. After the wound was naturally dried, 10 μL of a 1 × 10^5^ spores mL^−1^ spore suspension of *B. cinerea* was inoculated onto the wound and allowed to dry naturally. Ten strawberries with artificial inoculation were placed onto a 15 cm glass plate. An NYDA plate coated with 500 μL of yeast cell suspension (1 × 10^8^ cells mL^−1^) was laid upside down on the glass plate. Another NYDA plate spread with 500 μL of sterile distilled water was used as a control. The plates were sealed with two layers of parafilm to create a closed environment and stored at 90% relative humidity and 20 °C for 4 d. After four days of storage, the sealing film was torn off, leaving a small gap for ventilation. The strawberries were further stored for two more days. On the 6th day of storage, the disease incidence rate (percentage of infected strawberries) and lesion diameter were measured. The experiment was repeated three times.

### 2.5. Microscopic Observation of Hyphae Morphology and Ultrastructure

*B. cinerea* hyphae were exposed to VOCs of *S. spartinae* W9, as described in 2.3. After incubation for five days, the mycelium block of the *B. cinerea* culture was removed and fixed in 2.5% glutaraldehyde in 0.1 M PBS (pH 7.2) overnight at 4 °C. For the hyphae morphology observation, further experimental procedures were conducted following the method described in Xu et al. [15]. The fixed samples were first rinsed with 0.1 M PBS (pH 7.2) three times, each for 15 min. Then, they were dehydrated using ethanol gradients (30%, 50%, 70%, 80%, 90%) for 15 min at each concentration. Absolute ethanol was used for dehydration twice, also for 15 min each time. The samples were further dehydrated with anhydrous ethanol: tert-butanol mixture (in ratio of 3:1, 1:1, 3:1) for 15 min each. Pure tert-butanol was then replaced for 20 min. After centrifugation, the supernatant was removed, and an appropriate amount of tert-butanol was added. The sample was then placed in a freeze-dryer for further drying. Finally, the samples were observed using scanning electron microscopy (SEM) (Hitachi S-3400N, Tokyo, Japan) after coating with a thin layer of gold. 

For ultrastructure observing, the samples were prepared and observed following the method described by Shao et al. [16]. Briefly, the fixed samples were washed three times in PBS (pH 7.2, 0.1 M) to remove any residual fixative. The washed samples were further post-fixed with 1% osmium tetroxide for 2 h, then rinsed three times with PBS, and dehydrated using a series of dehydration solution gradients (30%, 50%, 70%, 90%), each time for 15 min. The samples were then dehydrated with 90% acetone once, followed by four dehydrations with anhydrous acetone, each time for 15 min. After embedding, polymerization, sectioning, and double staining with uranyl acetate and lead citrate, the sections were finally observed using transmission electron microscopy (TEM) (Hitachi H-7650, Tokyo, Japan).

### 2.6. Identification of VOC Components

The preparation method of yeast VOC samples was slightly modified according to Yalage Don et al. [17]. A 20 mL glass extraction bottle was sterilized by autoclaving, and the bottle cap was sterilized using ultraviolet light for 2 h. Five mL of sterile NYDA medium was pipetted into the sterilized glass extraction bottle to create a ramp. A cell suspension of *S. spartinae* W9 (1 × 10^8^ cells mL^−1^, 30 μL) was transferred onto the ramp. The suspension was spread evenly on the ramp surface. The glass extraction bottle was sealed and then incubated at 28 °C for 48 h. The control group consisted of a glass extraction bottle inoculated with an equal volume of sterile distilled water instead of the yeast suspension. After the cultivation, the VOCs produced by *S. spartinae* W9 were detected using headspace solid-phase microextraction gas chromatography–mass spectrometry (HS-SPME/GC-MS), according to a previous method [14].

The extraction and determination process of volatile components was as follows: the sample was first shaken and equilibrated at 50 °C for 30 min, and then, VOCs were extracted by the SPME fiber with 50/30 μm, DVB/CAR/PDMS (Supelco Co., Bellefonte, PA, USA;) coating for 30 min at 50 °C. Once adsorption was completed, the SPME fiber was inserted into the GC (7890B–7000C, Agilent Technologies Inc., Santa Clara, CA, USA) inlet and desorbed at 250 °C for 7 min. A DB-5 chromatographic column (30 m × 0.25 mm × 0.25 μm) was utilized to separate the VOC samples, and the separation program was set as follows: 35 °C for 2 min; the temperature was then increased to 100 °C at a rate of 5 °C min^−1^ and held for 1 min. Next, the temperature was further increased to 230 °C at a rate of 7 °C min^−1^ and kept for 12 min. The mass spectrometer utilized a 70 eV EI ion source, with a filament emission current of 200 μA and an ion source temperature of 230 °C. The scanning mass range was set as 29–500 m/z. The n-alkanes (C_7_ to C_40_, o2si, Charleston, SC, USA) were run using the same conditions to calculate [18]. The compositions of VOCs were identified by comparing the calculated LRI with the literature LRI and the mass spectrum in the NIST spectral library. Components that were only produced or increased in the treatment group were considered VOCs produced by *S. spartinae* W9. Each treatment consisted of five biological replicates.

### 2.7. Effect of Selected Individual VOCs on Mycelial Growth of B. cinerea

The antifungal activity of four main single components of yeast VOCs was evaluated following the method described by Lyu et al. [19]. 3-methyl-1-butanol (AR, ≥98.5%), 2-methyl-1-butanol (AR, ≥98%), 2-phenylethanol (AR, ≥99%), isoamyl acetate (AR, ≥99%) were purchased from Shanghai Macklin Biochemical Technology Co., Ltd. (Shanghai, China). A mycelium block with a diameter of 6 mm was taken from the edge of a 5-day-old *B. cinerea* culture and positioned at the center of a PDA plate. A sterile filter paper with a 3 cm diameter was placed on the bottom of the petri dish, and a specific volume of 3-methyl-1-butanol, 2-methyl-1-butanol, 2-phenylethanol, isoamyl acetate was dropped onto the filter paper, resulting in fumigation concentrations of 50, 100, and 150 μL L^−1^, respectively. As a control, 100 μL of sterile water was added to the filter paper. The plate with *B. cinerea* block was placed on top of the cover plate with filter paper, and the petri dish was sealed with two layers of parafilm. The sealed plate was then incubated at 25 °C for 5 d. The diameter of *B. cinerea* colony was measured daily, and the inhibition rate was calculated. Each treatment was replicated three times, and the experiment was repeated twice.

### 2.8. Statistical Analysis

Data analysis was performed using SPSS (version 22.0, SPSS Inc., Chicago, IL, USA). The data are presented as mean ± standard deviation (SD). The differences between the data were assessed using Duncan’s multiple range tests of one-way analysis of variance (ANOVA), or an independent samples *t*-test. A significance level of *p* < 0.05 was used to indicate the data were significantly different. 

## 3. Results

### 3.1. VOCs Produced by S. spartinae W9 Inhibited Mycelium Growth and Spore Germination of B. cinerea

**Figure 1:**
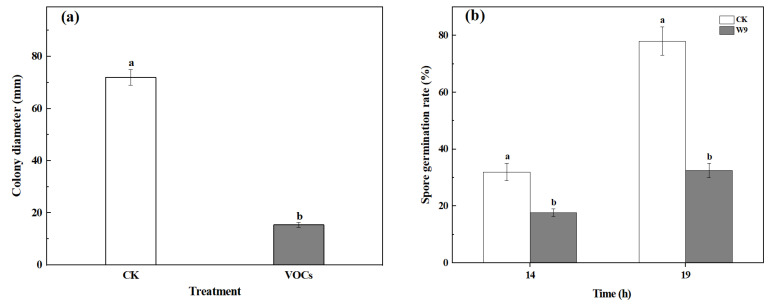
Antifungal effects of VOCs emitted by *S. spartinae* W9 on *B. cinerea*. (**a**) Mycelium growth of *B. cinerea*. The hyphae were cultured in PDA at 25 °C for 5 d under control and yeast VOCs treatment conditions. (**b**) Spore germination rate of *B. cinerea*. The spore suspensions were treated under control and yeast VOCs treatment conditions, and incubated at 25 °C for 14~19 h, respectively. Columns with different letters are significantly different according to an independent samples t-test at *p* < 0.05.

After five days of cultivation, the colony diameter of the *S. spartinae* W9 VOCs treatment group was 16.0 mm, which was 77.8% smaller than that of the control group, demonstrating a significant difference (*p* < 0.05) (Figure 1a). As shown in Figure 1b, the germination rate of the VOCs-treated group was significantly lower than that of the control group after 14 h and 19 h of culture (*p* < 0.05). When the incubation time reached 19 h, the spore germination rates of the control group and the VOCs treatment group were 78.0% and 32.5%, respectively, indicating a 58.3% reduction compared to the control treatment. These indicate that the VOCs produced by *S. spartinae* W9 inhibit the mycelial growth and spore germination of *B. cinerea*.

### 3.2. VOCs Produced by S. spartinae W9 Suppressed Strawberry Gray Mold

**Figure 2:**
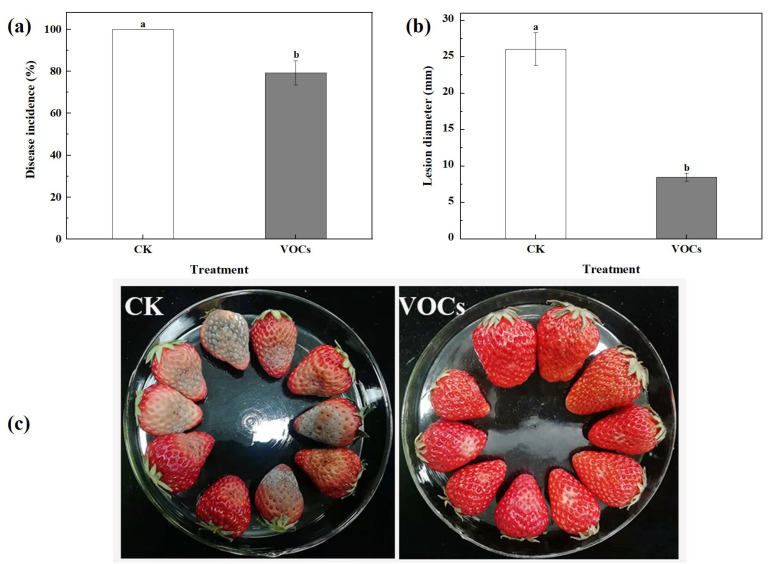
Biocontrol effects of gray mold by VOCs produced from *S. spartinae* W9 on artificially infected strawberry fruits after 6 d of storage. Disease incidence (**a**), lesion diameter (**b**), and pictures of representative strawberry samples recorded on the sixth day of storage (**c**). Columns with different letters are significantly different according to an independent samples t-test at *p* < 0.05.

As shown in Figure 2, after 6 d of storage, the yeast VOCs-treated group exhibited a significantly lower incidence rate and lesion diameter compared to the control group (*p* < 0.05). The control group had a 100% incidence rate and a lesion diameter of 26.1 mm, whereas the VOCs treatment group showed an incidence rate of 79.3% and a lesion diameter of 8.5 mm, making a reduction of 20.7% and 67.4%, respectively, compared to the control group. These data indicate that the VOCs produced by *S. spartinae* W9 effectively inhibit the occurrence and development of strawberry gray mold, and VOCs serve as a vital biocontrol mechanism of *S. spartinae* W9.

### 3.3. VOCs produced by S. spartinae W9 Destroyed the Mycelium Surface Morphology of B. cinerea

**Figure 3:**
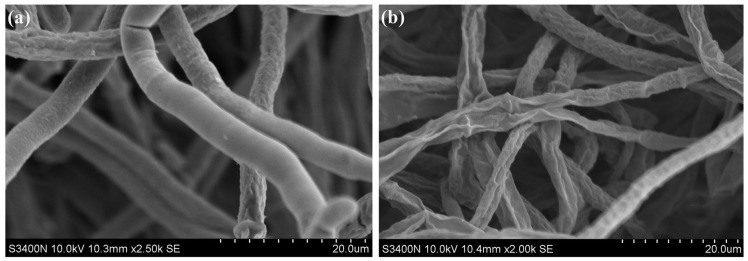
Scanning electron microscope observation of the hyphae morphology of *B. cinerea* treated with VOCs produced from *S. spartinae* W9. (**a**) Control group; (**b**) VOCs treatment group. The hyphae were cultured in PDA at 25 ℃ for 5 d under control and yeast VOCs treatment, respectively. Magnification: ×2500 (**a**); ×2000 (**b**).

After being cultured at 25 °C for 5 d, the hyphae of the control group appeared rough, with smooth and plump surface morphology, while some hyphae exhibited slight wrinkles (Figure 3a). In contrast, the mycelia of *B. cinerea* treated with VOCs from *S. spartinae* W9 were smaller and displayed more severe surface shriveling than the control (Figure 3b). These observations show that the VOCs produced by *S. spartinae* W9 cause damage [17] to the mycelial morphology of *B. cinerea*.

### 3.4. VOCs Produced by S. spartinae W9 Damaged the Cell Ultrastructure of B. cinerea

**Figure 4:**
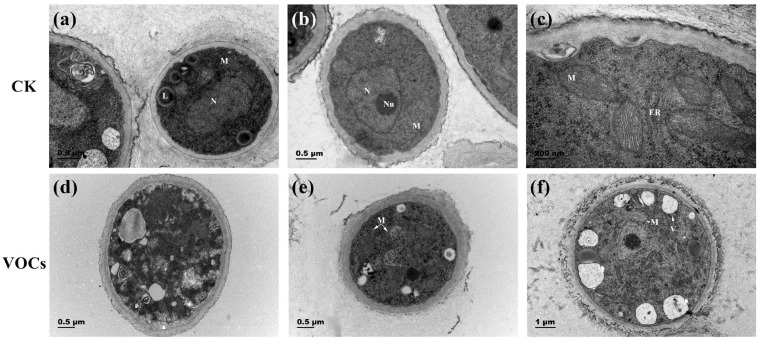
Transmission electron microscope observation of *B. cinerea* hyphae ultrastructure treated with VOCs produced by *S. spartinae* W9. Images (**a**–**c**) represent the control group, while images (**d**–**f**) represent the yeast VOCs treatment group. The arrow in (**d**) indicates slight plasmolysis. L: lipid body; M: mitochondria; N: nuclei; Nu: nucleus; ER: endoplasmic reticulum; V: vacuole. Magnification: ×30,000 (**a**,**b**); ×80,000 (**c**); ×25,000 (**d**,**e**); ×15,000 (**f**).

TEM observations revealed differences in the hyphae cell walls between the control group and the *S. spartinae* W9 VOCs-treated group. In the control group, the hyphae exhibited clear boundaries, and organelles such as liposomes, mitochondria, nuclei, and nucleoli were clearly visible. Additionally, the presence of few small vacuoles was observed (Figure 4a–c). Conversely, in the *S. spartinae* W9 VOCs-treated group, the hyphal cells formed larger vacuoles, some cells showed slight plasmolysis (Figure 4d), mitochondria appeared swelled (Figure 4e,f), and the matrix electron density decreased (Figure 4f). These observations indicate that the VOCs produced by *S. spartinae* W9 induce damage in the internal structure of *B. cinerea* hyphae.

### 3.5. Composition of VOCs Produced by S. spartinae W9

As shown in Table 1, *S. spartinae* W9 produced 18 main VOCs, including 4 alcohols, 4 esters, 3 heterocycles, 2 alkanes, 1 acid, 1 amide, 1 amine, 1 benzene, and 1 ether. Among them, 3-methyl-1-butanol exhibited the highest abundance, with a relative peak area of 51.29%. This was followed by 2-methyl-1-butanol (11.74%), 2-phenylethanol (9.02%), formamide (8.40%), dimethyl ether (5.26%), isoamyl acetate (4.32%), dimethylamine (2.90%), formic acid (1.44%), and ethyl hexanoate (1.07%). The remaining VOCs had a relative peak area of less than 1.0%. 

### 3.6. Antifungal Effect of Main VOCs Produced by S. spartinae W9

3-methyl-1-butanol, 2-methyl-1-butanol, 2-phenylethanol, and isoamyl acetate from the main VOCs of *S. spartinae* W9 were investigated for their antifungal effects on *B. cinerea* mycelium growth. As shown in Table 2, all these pure substances exhibited varying inhibitory effects on the growth of *B. cinerea* mycelia. The inhibition rate increased with the increase in the concentration of the substances and decreased with the increase in the incubation time. On the second day, the inhibitory rates of the pure compounds at a concentration of 150 μL L^−1^ on mycelial growth ranged from 49% to 100%. On the fifth day, the inhibitory rates of the compound at the same concentration ranged from 21% to 92%. Notably, 2-phenylethanol showed the most potent antifungal effect, with an inhibition rate of 100% on the second day and 92.9% on the fifth day of incubation. These were significantly higher than the inhibition rates observed in other treatment groups (*p* < 0.05). These findings indicate that the main VOCs produced by *S. spartinae* W9 possess antifungal activity against the mycelium growth of *B. cinerea*, with 2-phenylethanol exhibiting the strongest inhibition effect.

## 4. Discussion

The VOCs with antimicrobial characteristics have been documented in bacteria [19,20,21], yeasts [22,23,24], and filamentous fungi [25,26]. The release of VOCs has emerged as a critical mode of action of biocontrol microorganisms. With the trait of being volatile and rarely leaving residue, applying antagonistic microorganism VOC products in a closed non-contact environment is a new field of biological control applications [9]. In addition to exerting direct antimicrobial activity, microbial VOCs may play a role in complex intra- and inter-species interactions, such as intercellular communication, cell differentiation, morphogenesis, plant growth promotion, and induction of plant resistance [9,10,27].

It is indeed true that not all VOCs produced by yeasts have antifungal effects. While the literature has reported that certain VOCs from yeasts exhibit antimicrobial activity, the antifungal effects can vary among different species and strains. For instance, a study conducted in Thailand isolated 366 yeast strains from field crops, of which only 49 strains exhibited antifungal ability against the mycelium growth of *Aspergillus* spp., and the degree of fungal growth inhibition also varied among different species [28]. Some specific examples of yeasts and their VOCs with antifungal effects include: *A. pullulans* [7,17], *Hanseniaspora uvarum* [23,29], *Hanseniaspora opuntiae* [30], *Candida sake* [22], *Yarrowia lipolytica*, *Kluyveromyces marxianus* and *Pichia kudriavzevii* [31], and *Wickerhamomyces anomalus*, *Metschnikowia pulcherrima* and *Saccharomyces cerevisiae* [4]. These yeast VOCs have been reported to exhibit antifungal effects against various fungal species, including *Pestalotiopsis vismiae*, *B. cinerea*, *Aspergillus* spp., *Penicillium spp.*, *Monilinia spp.*, and *Alternaria alternata.*

In the present study, the strong antifungal activity of VOCs produced by *S. spartinae* W9 on the mycelium growth and spore germination of *B. cinerea* in vitro (Figure 1) was observed and demonstrated, as well as a decrease in disease incidence and the lesion diameter of gray mold in strawberry fruit (Figure 2). This may be the first report describing the fumigation of *S. spartinae* VOCs to suppress the growth of gray mold in strawberry fruit. Similar results have been reported previously; for instance, VOCs produced by *H. uvarum* [23,29], *W. anomalus*, *M. pulcherrima*, and *S. cerevisiae* [4] reduced *B. cinerea* growth in vitro and strawberry gray mold incidence, and even prolonged the storage time and shelf life of the fruit [23]. The present study confirmed that *S. spartinae* W9 VOCs damaged the morphology and internal structure of *B. cinerea* hyphae (Figure 3 and Figure 4). These results are consistent with those of Farbo et al. [3], who found that yeast VOCs emitted by *Lachancea thermotolerans* caused hyphal swollen and lysis in *Aspergillus* spp. The volatiles emitted by *S. cerevisiae* resulted in hyphal deformities, damage, and reduced size in *Aspergillus flavus* [32]. Xing et al. [20] also discovered that VOCs of *Streptomyces fimicarius* caused morphological disruption in hyphae, sporangiophores, and sporangia of *Peronophythora litchi*, and even led to thickened cell walls and cell membranes, as well as damage in vacuoles and mitochondria.

Using the HS-SPME/GC-MS technology, we identified 18 main VOCs produced by *S. spartinae* W9. The top three VOCs in terms of relative content were 3-methyl-1-butanol, 2-methyl-1-butanol, and 2-phenylethanol, accounting for more than 72% of the total VOCs (Table 1). These VOCs, along with isoamyl acetate, are commonly found in yeast [33,34,35]. Di Francesco et al. [33] studied the effects of the main volatile substances produced by *A. pullulans* and found that 2-phenylethanol, 3-methyl-1-butanol, and 2-methyl-1-butanol inhibited the spore germination of *B. cinerea*. Among them, 2-phenylethanol exhibited the strongest inhibitory effect, with an EC_50_ of 0.57 μL mL^−1^. Ando et al. [36] demonstrated that isoamyl acetate and 3-methyl-1-butanol inhibited the germination of spores from 12 and 15 different filamentous fungi, respectively. Similar results were also observed in the present study regarding the inhibitory effects of four VOCs produced by *S. spartinae* W9, namely 3-methyl-1-butanol, 2-methyl-1-butanol, 2-phenylethanol, and isoamyl acetate, on the hyphal growth of *B. cinerea*. Among them, 2-phenylethanol showed the strongest antifungal effect (Table 2). 

In recent years, there has been research interest in 2-phenylethanol, a common VOC secreted by many yeasts. Hua et al. [37] reported that 2-phenylethanol is the main volatile substance emitted by *Pichia anomala*. They found it inhibited mycelium growth, conidia germination, toxin production, and aflatoxin gene expression in *A. flauvs*. Yalage Don et al. [17] discovered that ethanol and 2-phenylethanol are key substances of VOCs emitted by *A. pullulans*. These substances inhibited the fungal growth of *B. cinerea* and *A. alternata*. Lu et al. [38] revealed that 2-phenylethanol is the third-most abundant component in the VOCs of *Candida quercitrusa*. They also found that it blocked the oxidative phosphorylation pathway of *Phytophthora infestans*. Zou et al. [14] also identified 2-phenylethanol as the key compound in the VOCs of *Candida pseudolambica* W16, and observed that 2-phenylethanol inhibited *B. cinerea* growth by causing cell membrane damage and inducing reactive oxygen species (ROS) stress [39]. 

Based on the findings of this paper, we speculate the production of several antifungal volatile substances may serve as a crucial biocontrol mechanism of *S. spartinae* W9. The antifungal performance of the yeast VOCs is probably due to an additive and synergistic action of several compounds, not only the above four compounds, but also other volatile components with lower concentrations. The mixtures of VOCs from *S. spartinae* W9 possibly elicit multicomponent modes of action, such as cell membrane permeability and disruption, organelle damage, electrolyte loss, and oxidative stress [20,34]. The profound cytological impact implies that VOCs from *S. spartinae* W9 cause significant changes in gene expression in *B. cinerea*, ultimately inhibiting its growth. The detailed antifungal mechanisms of *S. spartinae* W9 VOCs need to be further explored. 

## 5. Conclusions

In conclusion, *S. spartinae* W9 produced 18 main VOCs, some of which exhibited antifungal activities, resulting in microscopic damage to *B. cinerea* and displaying biocontrol ability in vitro and in strawberry fruits during storage. The VOCs produced by *S. spartinae* W9 offer a promising biocontrol strategy against gray mold in strawberries. For example, fumigation with yeast VOCs or specific antifungal volatile compounds. However, further investigations are required to explore the synergistic effects of these VOCs and their broad-spectrum antimicrobial effects on other microorganisms. Additionally, practical applications of these VOCs need to be explored. 

## Figures and Tables

**Table 1:** List of major VOCs produced by *S. spartinae* W9 after 48 h of incubation on NYDA medium at 28 °C.

Volatile Compound	CAS	Molecular Formula	RT ^a^ (min)	RA ^b^ (%)	LRI_(calculated)_^c^	LRI_(lit)_^d^
*Alcohols*						
3-Methyl-1-butanol	123-51-3	C_5_H_12_O	4.223	51.29	726	727
2-Methyl-1-butanol	137-32-6	C_5_H_12_O	4.611	11.74	742	743
2-phenylethanol	60-12-8	C_8_H_10_O	17.016	9.02	1110	1110
3-Cyclohexen-1-ol	822-66-2	C_6_H_10_O	19.557	0.44	1179	1182
*Esters*						
Isoamyl acetate	123-92-2	C_7_H_14_O_2_	8.321	4.32	877	878
Ethyl hexanoate	123-66-0	C_8_H_16_O_2_	12.796	1.07	999	999
Octanoic acid, ethyl ester	106-32-1	C_10_H_20_O_2_	20.185	0.50	1196	1193
Decanoic acid, ethyl ester	110-38-3	C_12_H_24_O_2_	26.941	0.88	1394	1391
*Heterocycles*						
Furan, 2-pentyl-	3777-69-3	C_9_H_14_O	12.393	0.41	988	989
Pyrazine, 3-ethyl-2,5-dimethyl-	13360-65-1	C_8_H_12_N_2_	15.629	0.63	1074	1077
Pyrazine, 2, 5-dimethyl-3-(3-methylbutyl)-	18433-98-2	C_11_H_18_N_2_	24.177	0.29	1310	1310
*Alkanes*						
Pentane, 3-ethyl-	617-78-7	C_7_H_16_	3.598	0.55	696	687
Dodecane	112-40-3	C_12_H_26_	20.334	0.37	1200	1200
*Acids*						
Formic acid	64-18-6	CH_2_O_2_	2.315	1.44	-^e^	-
*Amides*						
Formamide	75-12-7	CH_3_NO	2.079	8.40	-	-
*Amines*						
Dimethylamine	124-40-3	C_2_H_7_N	2.139	2.90	-	426
*Benzenes*						
o-Cymene	527-84-4	C_10_H_14_	13.722	0.49	1023	1027
*Ethers*						
Dimethyl ether	115-10-6	C_2_H_6_O	1.755	5.26	-	-

^a^ RT: Retention time. ^b^ RA: Relative peak area. ^c^ LRI_(calculated)_: Linear retention index, calculated via analysis of n-alkanes. ^d^ LRI_(lit)_: Linear retention index from NIST Standard Reference Database. ^e^ “-”: the LRI of the compound was either not calculated or found in the NIST database.

**Table 2:** Effects of selected individual of VOCs produced by *S. spartinae* W9 on mycelial growth of *B. cinerea*.

Volatile Compound	Inhibition Rate (%)					
	Day 2			Day 5		
	50 μL/L	100 μL/L	150 μL/L	50 μL/L	100 μL/L	150 μL/L
3-Methyl-1-butanol	25.6 ± 12.3 ^c^	43.2 ± 3.7 ^c^	49.1 ± 9.4 ^cd^	4.3 ± 4.5 ^b^	14.4 ± 5.2 ^b^	21.4 ± 8.2 ^c^
2-Methyl-1-butanol	29.2 ± 6.1 ^bc^	49.9 ± 3.2 ^bc^	56.7 ± 3.3 ^c^	5.7 ± 6.4 ^b^	19.7 ± 8.4 ^b^	27.4 ± 6.4 ^bc^
2-Phenylethanol	76.6 ± 13.4 ^a^	87.4 ± 13.4 ^a^	100 ± 0.0 ^a^	22.5 ± 11.6 ^a^	83.4 ± 2.7 ^a^	92.9 ± 7.9 ^a^
Isoamyl acetate	40.3 ± 2.8 ^b^	53.9 ± 5.4 ^b^	90.7 ± 7.2 ^b^	17.2 ± 1.3 ^a^	22.1 ± 6.4 ^b^	25.5 ± 4.9 ^c^

Note: Different alphabetic superscripts in the same column indicate significant difference (*p* < 0.05) based on Independent Samples *t*-test or Duncan’s Multiple Range test.

## Data Availability

The data presented in this study are included in the article. Further inquiries can be directed to the corresponding author.
